# Retraction Note: JNK confers 5-fluorouracil resistance in p53-deficient and mutant p53-expressing colon cancer cells by inducing survival autophagy

**DOI:** 10.1038/s41598-021-82870-5

**Published:** 2021-02-09

**Authors:** Xinbing Sui, Na Kong, Xian Wang, Yong Fang, Xiaotong Hu, Yinghua Xu, Wei Chen, Kaifeng Wang, Da Li, Wei Jin, Fang Lou, Yu Zheng, Hong Hu, Liu Gong, Xiaoyun Zhou, Hongming Pan, Weidong Han

**Affiliations:** 1 Department of Medical Oncology, Sir Run Run Shaw Hospital, Zhejiang University, Hangzhou, China; 2 Biomedical Research Center and Key Laboratory of Biotherapy of Zhejiang Province, Hangzhou, 310016 China

Retraction of: *Scientific Reports* 10.1038/srep04694, published online 15 April 2014

The Editors have retracted this Article. Concerns were raised regarding a number of figures, specifically:

- Figure 1B: the HCT116 p53+/+ GAPDH band appears to be identical to the HT29 GADPH band for Figure 4A

- Figure 1B: the hCT116 p53−/− GADPH band appears to be identical to the HCT116 p53+/+ GADPH band for Figure 4A

- Figure 4A: the HCT116 p53+/+ p-JNK band appears to be identical to the band for RKO JNK

- Figure 4B: the HCT116p53−/− control 5-FU(20mu) GAPDH band appears to be identical to the HT29 GAPDH band in Figure 5B

The data reported in this Article are therefore unreliable.

The Authors disagree with the retraction.

